# Yi-Gan San Restores Behavioral Alterations and a Decrease of Brain Glutathione Level in a Mouse Model of Schizophrenia

**DOI:** 10.4137/jcnsd.s2255

**Published:** 2009-02-12

**Authors:** Manabu Makinodan, Takahira Yamauchi, Kouko Tatsumi, Hiroaki Okuda, Yoshinobu Noriyama, Miyuki Sadamatsu, Toshifumi Kishimoto, Akio Wanaka

**Affiliations:** 1 Department of Psychiatry, Nara Medical University Faculty of Medicine.; 2 Department of Anatomy and Neuroscience, Nara Medical University Faculty of Medicine

**Keywords:** Yi-gan san (yokukansan), schizophrenia, open field test, prepulse inhibition, cognitive deficits, glutathione

## Abstract

The traditional Chinese herbal medicine yi-gan san has been used to cure neuropsychological disorders. Schizophrenia can be one of the target diseases of yi-gan san. We aimed at evaluating the possible use of yi-gan san in improving the schizophrenic symptoms of an animal model. Yi-gan san or distilled water was administered to mice born from pregnant mice injected with polyinosinic-polycytidilic acid or phosphate buffered saline. The former is a model of schizophrenia based on the epidemiological data that maternal infection leads to psychotic disorders including schizophrenia in the offspring. Prepulse inhibition and sensitivity to methamphetamine in open field tests were analyzed and the total glutathione content of whole brains was measured. Yi-gan san reversed the decrease in prepulse inhibition, hypersensitivity to methamphetamine and cognitive deficits found in the model mice to the level of control mice. Total glutathione content in whole brains was reduced in the model mice but was restored to normal levels by yi-gan san treatment. These results suggest that yi-gan san may have ameliorating effects on the pathological symptoms of schizophrenia.

## Introduction

Yi-gan san (YGS, yokukan-san in Japanese) has been administered to children for the treatment of restlessness and agitation. Recent studies revealed that YGS is also useful in treating neuropsychological disorders such as behavioral and psychological symptoms of dementia (BPSD) in the elderly,^1^–^4^ a number of symptoms of borderline personality disorder,^5^ tardive dyskinesia and psychotic symptoms of schizophrenia.^6^,^7^

In the case of schizophrenia, many effective antipsychotics have been developed and widely used, but some of them induce drowsiness and extrapyramidal symptoms (EPS). YGS has only mild sedative effects and induces no EPS, making it a promising candidate as an antipsychotic for schizophrenia. In the present study, we examined the efficacy of YGS in treating a mouse model of schizophrenia, which is based on the epidemiological data that maternal infection leads to schizophrenia in offspring.^8^ Polyinosinicpolycytidilic acid (poly I:C) is commonly used to induce an immune response similar to that induced by viral infection.^9^ When early pregnant mice receive intraperitoneal poly I:C injection, the behavior of their pups (poly I:C-mice) becomes schizophrenic. The prepulse inhibition is decreased, the sensitivity to dopamine release is increased, and the cognitive function is impaired in poly I:C-mice.^10^–^13^ Several reports have shown that the abnormal behaviors were improved by antipsychotic treatments. In this model, we investigated whether YGS could restore impaired PPI, methamphetamine hypersensitivity in an open field test and cognitive deficits in a novel object recognition test (NORT). In addition, we measured total glutathione content (both reduced and oxidized species) in whole brain after oral administration of YGS to poly I:C-mice, since the cellular glutathione level is linked to the pathogenesis of schizophrenia^14^ and the brain and cerebrospinal fluid glutathione contents were decreased in schizophrenic patients.^15^

## Material and Methods

### Animals, prenatal treatment and YGS administration

C57BL/6 mice were mated at about 3 months of age and the first day after copulation was defined as embryonic day 0 (E0). Pregnant mice received either a single i.p. injection of poly I:C (20 mg/kg,^13^) dissolved in phosphate buffered saline (PBS) or an equivalent volume of PBS at embryonic day 12.5 (E12.5). Pups born from poly I:C-treated mice and PBS-treated mice are hereafter referred to as poly I:C-mice and PBS-mice, respectively. Pups were weaned and housed 4 to a cage according to sex and litter at postnatal day 21 (P21), and housed in a temperature- and humidity-controlled animal facility under a reversed light-dark cycle (lights on 8:00~20:00). Poly I:C-mice and PBS-mice were each divided into two groups containing twelve pups from three different litters. The three groups of pups were designated as PBS-control, poly I:C-control, and poly I:C-YGS groups. YGS suspended in distilled water (DW) (1 g/kg, 0.5 ml/day × 21 days; from P56 to P76) was administrated using a syringe with a metal feeding tube to poly I:C-YGS groups and the same amount of DW was given to control pups on the same schedule. Locomotor activity in an open field test and PPI were measured at P77. For the biochemical analysis of total glutathione content (see below), three other groups of pups (four pups in each group) were treated as above (YGS group and control group) and whole brains were removed at P77. All animals were male and maintained with food and water ad lib through the duration of experiments. Experimental protocols were according to the guidelines of the Animal Care Committee of Nara Medical University and were in accordance with the policies established in the NIH Guide for the Care and Use of Laboratory Animals.

### Prepulse inhibition test

The mouse was placed in a translucent acrylic cage (7 cm × 7 cm × 16.5 cm). Movements of animals were detected by a piezoelectric accelerometer (GH313A, GA245SO: Keyence, Kyoto, Japan) attached to the bottom of the cage. White noise at 115 dB for duration of 50 msec was used as the acoustic startle stimulus (pulse). A noise prepulse of 85 dB was presented for 30 msec. Background noise was kept at a relatively constant level, 70–73 dB. The test session consisted of a total of 19 trials: 10 startle trials without a prepulse (habituation), followed by 9 trials of prepulse test session. The mean inter-trial interval was 25 sec (range, 15–45 sec). In the prepulse trials, prepulse with a lead time of 50 msec was followed by the pulse. Four pulse-alone trials and five prepulse trials were presented in random order. Relative startle response (RSR) was calculated using the formula RSR = (1-PP/N) × 100, where PP was designated as the mean response with prepulse and N was designated as the mean response without a prepulse. (n = 12, PBS-micecontrol; n = 12, poly I:C-mice-control; n = 12, poly I:C-mice-YGS).

### Open field test

The open field consists of a square acrylic box (40 × 40 cm) and a video camera for recording locomotion of mice. For monitoring of locomotor activity in a novel environment (novel test), mice were placed in a novel open field for 10 min, and the successive activity in the same field during the following 10 min was measured as basic locomotor activity. Next, the locomotor activity was monitored for 10 min in the same open field starting 30 min after the injection of methamphetamine (1 mg/kg). We then analyzed the video-recorded locomotion using tracking software, TopScan Suite (Clever Sys Inc.) (n = 12, PBS-mice-control; n = 12, poly I:C-mice-control; n = 12, poly I:C-mice-YGS).

### Novel object recognition test (NORT)

In a session, two objects, which are different in their shape and color, but similar in size, were placed diametrically opposite each other apart from the corner (18 cm) in a familiar box (40 × 40 × 40 cm). Before the session, mice were habituated in the box for 3 days. Each mouse was allowed to explore in the box for 10 min. The mice were considered to be exploring the object when the nose, not the body, was within 2 cm from the edge of the object. The time spent exploring each object was recorded using TopScan Suite (Clever Sys Inc.,). After the training, the mice were returned to their home cages, and the box and objects were cleaned with ethanol to avoid any effects of odors. The mice were allowed to explore for 5 min in the same box with one novel object instead of one object used in the training session 24 h after the termination of the training session. The time spent exploring each object was recorded as described above. The ratio of time spent exploring any one of the two objects (training session) or the novel object (retention session) to the total time spent exploring both objects was employed for the measure of memory function. (n = 11, PBS-mice-control; n = 10, poly I:C-mice-control; n = 9, poly I:C-mice-YGS).

### Total glutathione assay

The whole brains of PBS-mice with DW, poly I:C-mice with DW and poly I:C-mice with YGS were removed. Brains were homogenized in 5% sulphosalicylic acid (0.5 mg/ml) and centrifuged at 8000 × g for 10 min. The supernatant was assayed using a total glutathione quantification kit (Dojin Molecular Technologies Inc., Japan) according to instructions provided by the manufacturer. Briefly, the total glutathione content was detected by measuring the optic density of samples and glutathione standard solutions. (n = 4, PBS-mice- control; n = 4, poly I:C-mice-control; n = 4, poly I:C-mice-YGS). Reduced glutathione and 2-nitrobenzoic acid in the kit react to generate 2-nitro-5-thiobenzoic acid and glutathione disulfide. Since 2-nitro-5-thiobenzoic acid is a yellow colored product, glutathione concentration in a sample solution can be determined by the measurement at 412 nm absorbance. Reduced glutathione is generated from 2-nitro-5-thiobenzoic acid by glutathione reductase, and reacts with 2-nitrobenzoic acid again to produce 2-nitro-5-thiobenzoic acid. Therefore, this recycling reaction improves the sensitivity to total glutathione detection.

### Statistical analysis

Bonferroni’s test was used to determine the significant differences. Values of p < 0.05 were considered to be statistically significant.

## Results

### YGS restored a decrease in prepulse inhibition of poly I:C-mice

**Figure 1:**
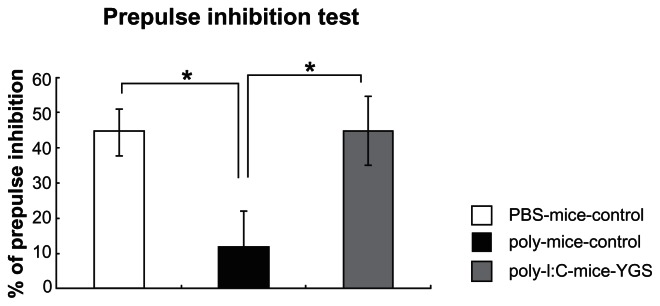
Prepulse inhibition test. Prepulse inhibition was decreased in poly I:C-control mice compared to PBS-control mice (*; p < 0.05). YGS reversed the disruption of prepulse inhibition in poly I:C-control mice to the level of PBS-control mice (*; p < 0.05).

PPI was decreased in the offspring born from poly I:C-injected mice (poly I:C-control group) compared with control mice (PBS-control group)(Fig. 1, p < 0.05). YGS administration (poly I:C-YGS group) significantly reversed the reduction in PPI in poly I:C-control mice (Fig. 1, p < 0.05) to a similar level as in PBS-control mice (Fig. 1, p < 0.05).

### YGS had no effect on basal activity, but prevented hyperlocomotion after methamphetamine injection in an open field test

**Figure 2:**
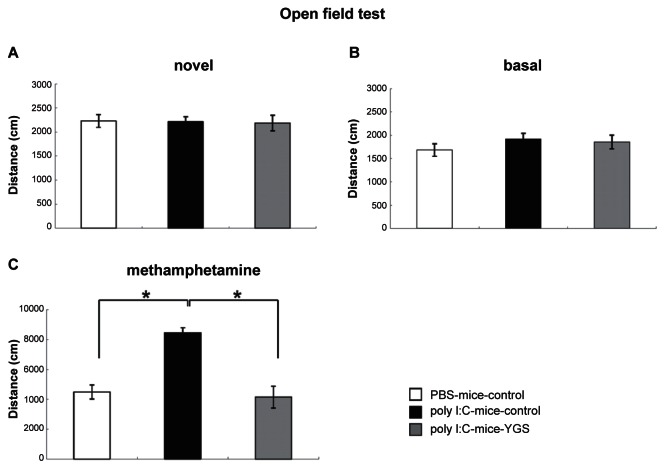
Open field test. Locomotor activity in the novel environment for the first 10 min was not different in PBS-control mice, poly I:C-control mice and poly I:C-YGS mice (**A**). In the next 10 min, the locomotor activity in the same field was also not different in the three groups (**B**). YGS attenuated the hyperactivity of poly I:C-control mice to the level of PBS-control mice in the same field for 10 min, 30 min after the injection of methamphetamine (**C**,*; p < 0.05).

In the novel open field, locomotor activity in PBS-control, poly I:C-control and poly I:C-YGS mice was not different (Fig. 2A, p < 0.05). Basal locomotor activity in the familiar open field was also not different in the three groups (Fig. 2B, p < 0.05). The activity of poly I:C-control mice was significantly higher than that of PBS-control mice after the injection of methamphetamine (Fig. 2C, p < 0.05). YGS administration significantly reversed the increased activity of poly I: C-control mice (Fig. 2C, p < 0.05) to a similar level to that of PBS-control mice (Fig. 2C, p < 0.05).

### YGS improved cognitive deficits of poly I:C-mice

**Figure 3:**
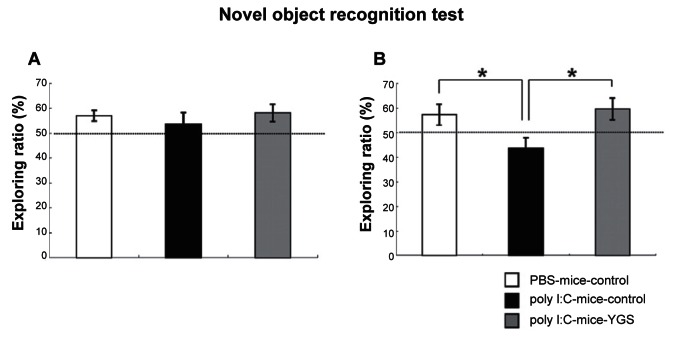
NORT. The rate of time spent exploring the two objects was not different in PBS-control, poly I:C-control and poly I:C-YGS mice in the training session (**A**). In the retention session, the rate of time exploring the novel object was lower in poly I:C-control mice than either PBS-control or poly I:C-YGS mice (**B**,*; p < 0.05).

Poly I:C-mice showed cognitive deficits in NORT as reported previously.^12^ The rate of time spent exploring the two objects was not different in PBS-control, poly I:C-control and poly I:C-YGS mice in the training session (Fig. 3A, p < 0.05). In the retention session, the rate of time exploring the novel object was lower in poly I:C-control mice than either PBS-control or poly I:C-YGS mice (Fig. 3B, p < 0.05, respectively). As Hashimoto et al. reported, the cognitive deficits with NORT might show negative symptoms, such as social withdrawal, that are related to cognitive deficits.^24^ Therefore, YGS might improve negative symptoms of schizophrenia.

### Total glutathione level was restored to a normal level by YGS treatment in poly I:C-mice

**Figure 4:**
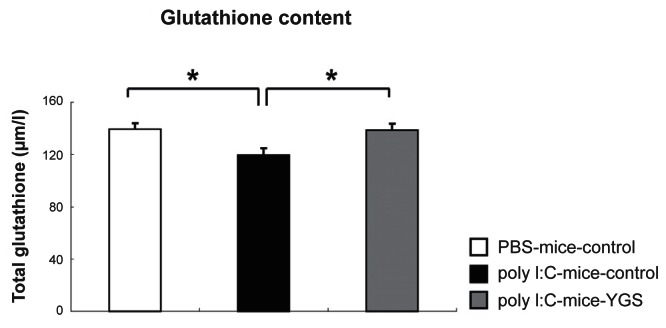
Total glutathione content of poly I:C-control mice was decreased compared to PBS-control mice (*; p < 0.05). YGS reversed the decrease in total glutathione content of poly I:C-control mice to the level of PBS-control mice (*; p < 0.05).

Glutathione is a small protein composed of three amino acids (cysteine, glutamate and glycine). Glutathione attenuates external or internal stress by scavenging free radicals and other reactive species and plays a critical role in defense against oxidative stress. Since glutathione levels were decreased in the medial prefrontal cortex and cerebrospinal fluid in schizophrenic patients,^15^ we analyzed total glutathione levels in PBS-control mice and poly I:C-control mice. The glutathione level was lower in the whole brain of poly I:C-control than PBS-control mice (Fig. 4, p < 0.05). The total glutathione level in poly I:C-YGS mice was significantly increased from that of poly I:C-control mice (Fig. 4, p < 0.05) to a comparable level to that of PBS-control mice (Fig. 4, p < 0.05).

## Discussion

YGS is a traditional herbal medicine and consists of seven different extracts, namely, Atractylodes lancea rhizome, Poria sclerotium, Cnidium rhizome, Uncaria thorn, Japanese Angelica root (Angelica radix), Bupleurum root and Glycyrrhiza in the proportions of 4:4:3:3:3:2:1.5.^16^ Liao et al. reported Angelicae radix exerts effects on γ-aminobutyric acid receptors and serotonin receptors.^17^ YGS is likely to have effects on not only BPSD of dementia, but also undesirable symptoms of borderline personality disorder and schizophrenia. Therefore, in the present study, we analyzed the effects on a mouse model of schizophrenia, that is poly I:C-mice.

Poly I:C-mice shows schizophrenia-like symptoms, decreased PPI and increased sensitivity to methamphetamine,^10^,^12^,^13^ but the basal locomotor activity is comparable to PBS-mice.^11^ In the present study, the locomotor activity of poly I:C-mice was also comparable to PBS-mice and YGS did not influence the activities of poly I:C-mice (Figs. 2A, 2B). This data is intriguing because the sedative effect of antipsychotics causes some clinical problems. The decreased PPI, increased sensitivity to methamphetamine and cognitive deficits in poly I:C-mice reverted to control levels after the administration of YGS (Figs. 1, 2, 3). These results suggest that YGS could have therapeutic effects equivalent to established antipsychotics without unwanted sedation in humans, as is the case with poly I:C-mice, although we have not examined whether YGS had sedative effect on PBS mice (control mice) in the present study. This point should be clarified before YGS is brought to clinical trial on schizophrenic patients.

Previous studies have shown that YGS attenuated 5-HT_2A_ receptor agonist-induced behavior^18^ and that dopamine receptors and serotonin receptors were involved in PPI.^19^–^22^ And, in the present study, YGS also improved cognitive deficits in NORT. Previously, Hashimoto et al. reported that phencyclidine-induced cognitive deficits in NORT were improved by subchronic administration of either clozapine or α7 nicotinic receptor agonist, but not of haloperidol.^23^,^24^ Although the mechanisms of the deficit and the improvement of cognitive functions in NORT are not known, it might be related to the function of NMDA receptor because the modulation of NMDA receptor functions could be involved in behavioral alterations of phencyclidine-injected mice and poly I:C-mice^25^ and the action mechanism of clozapine and α7 nicotinic receptor agonist.^24^,^26^,^27^ Additionally, a component of YGS, Uncaria rhynchophylla, has protective effect against apoptosis induced by N-methyl-D-aspartate in rat hippocampal slice.^28^ Therefore, the therapeutic effects of YGS on the abnormal behavior of poly I:C-mice could be achieved in part by modulating dopamine, serotonin or glutamate signaling pathways. Since YGS consists of multiple herbal extracts, further studies are required to clarify which compound(s) have therapeutic activities.

Glutathione levels were decreased in the medial prefrontal cortex and cerebrospinal fluid in schizophrenic patients^15^ and gene expression of glutathione-synthesizing enzymes and glutathione component in fibroblasts from schizophrenic patients was also decreased.^14^ In red blood cells from schizophrenic patients, antioxidant enzyme activities were decreased compared with the control group.^29^ Consistent with human studies mentioned above, we also found decreased glutathione levels in the whole brain of poly I:C-mice. YGS significantly restored the decreased total glutathione level (Fig. 3). As a glutathione precursor, N-acetyl-cysteine, improved mismatch negativity in schizophrenic patients^30^ and reversed working memory deficits caused by phencyclidine.^31^ The therapeutic effects of YGS observed in the present study may be, at least partly, attributed to recovered glutathione synthesis. It remains to be investigated whether upregulation of glutathione levels by a glutathione precursor could restore abnormal behaviors in poly I:C-mice.

The present study showed for the first time that Chinese herbal medicine, YGS, restores behavioral alterations and a decrease of brain glutathione level in schizophrenic model mice. These results suggest that YGS could be a novel remedy for the symptoms of schizophrenia.
